# Multidrug-Resistant *Aspergillus fumigatus* Carrying Mutations Linked to Environmental Fungicide Exposure — Three States, 2010–2017

**DOI:** 10.15585/mmwr.mm6738a5

**Published:** 2018-09-28

**Authors:** Karlyn D. Beer, Eileen C. Farnon, Seema Jain, Carol Jamerson, Sarah Lineberger, Jeffrey Miller, Elizabeth L. Berkow, Shawn R. Lockhart, Tom Chiller, Brendan R. Jackson

**Affiliations:** ^1^Division of Foodborne, Waterborne and Environmental Diseases, National Center for Emerging and Zoonotic Infectious Diseases, CDC; ^2^Philadelphia Department of Public Health; ^3^California Department of Public Health; ^4^Virginia Department of Health; ^5^Career Epidemiology Field Officer Program, CDC; ^6^Pennsylvania Department of Health.

The environmental mold *Aspergillus fumigatus* is the primary cause of invasive aspergillosis. In patients with high-risk conditions, including stem cell and organ transplant recipients, mortality exceeds 50%. Triazole antifungals have greatly improved survival (*1*); however, triazole-resistant *A. fumigatus* infections are increasingly reported worldwide and are associated with increased treatment failure and mortality (*2*). Of particular concern are resistant *A. fumigatus* isolates carrying either TR_34_/L98H or TR_46_/Y121F/T289A genetic resistance markers, which have been associated with environmental triazole fungicide use rather than previous patient exposure to antifungals (*3*,*4*). Reports of these triazole-resistant *A. fumigatus* strains have become common in Europe (*2*,*3*), but U.S. reports are limited (*5*). Because of the risk posed to immunocompromised patients, understanding the prevalence of such isolates in patients is important to guide clinical and public health decision-making. In 2011, CDC initiated passive laboratory monitoring for U.S. triazole-resistant *A. fumigatus* isolates through outreach to clinical laboratories. This system identified five TR_34_/L98H isolates collected from 2016 to 2017 (*6*), in addition to two other U.S. isolates collected in 2010 and 2014 and reported in 2015 (*5*). Four of these seven isolates were reported from Pennsylvania, two from Virginia, and one from California. Three isolates were collected from patients with invasive pulmonary aspergillosis, and four patients had no known previous triazole exposure. *A. fumigatus* resistant to all triazole medications is emerging in the United States, and clinicians and public health personnel need to be aware that resistant infections are possible even in patients not previously exposed to these medications.

Triazole antifungal medications are the primary treatment for invasive *A. fumigatus* infections, opportunistic infections that typically affect immunocompromised patients. Invasive aspergillosis is almost universally fatal without antifungal treatment. Clinical outcomes improved with the use of amphotericin B and have improved further with the introduction of mold-active triazole antifungals such as voriconazole, posaconazole, and itraconazole, which are also associated with fewer adverse events than is amphotericin B (*7*). Resistance to triazoles has been associated with treatment failure and increased mortality, but the prevalence of infection with resistant strains in U.S. hospitals is unknown (*1*,*4*). Structurally similar triazoles are used extensively as fungicides in agriculture and other environmental applications. *A. fumigatus* is not typically a plant pathogen but is common in soil and decaying plant material. Incidental exposure of *A. fumigatus* to fungicides during agricultural or other environmental applications can select for mutations conferring resistance to triazoles. *A. fumigatus* spores are known to be carried long distances in the air, putting patients at risk for infection with resistant strains, even in areas without known agricultural fungicide usage.

In Europe, molecular epidemiologic studies have identified two resistant *A. fumigatus* genotypes associated with environmental triazole exposure (*4*). These genotypes, TR_34_/L98H and TR_46_/Y121F/T289A, confer resistance to triazoles by altering the drug target, Cyp51A, which is involved in fungal cell wall synthesis. Importantly, TR_34_/L98H confers resistance to all mold-active medical triazoles without incurring a fitness cost or survival disadvantage to the fungus. *A. fumigatus* strains of this genotype have been isolated from the environment (e.g., compost, seeds, soil, commercial plant bulbs, and patient households) (*8*). Although these mutations have been detected repeatedly in environmental isolates, they have not been common among isolates from patients treated with long-term triazoles in whom resistance might have been expected to develop. Most (50%–75%) patients with TR_34_/L98H isolates have not been exposed to triazole therapy, further suggesting environmental acquisition of resistance (*3*).

Until 2015, no isolates with these genotypes had been reported in the United States; that year, a U.S. fungal reference laboratory reported detecting two TR_34_/L98H and two TR_46_/Y121F/T289A *A. fumigatus* isolates among 220 clinical isolates collected from 2001 to 2014 (*5*). In 2017, TR_34_/L98H *A. fumigatus* isolates were first detected in U.S. environmental samples obtained from a commercial peanut field treated with triazole fungicides (*9*). Together, these reports demonstrate that triazole-resistant *A. fumigatus* strains have emerged in the United States in both patients and the environment, likely caused by selection for resistance during environmental triazole use.

In 2011, CDC issued a request for clinical *A. fumigatus* isolates on the ClinMicroNet e-mail listserv of approximately 800 U.S. clinical microbiology laboratory directors, leading to a U.S. laboratory-based convenience sample of *A. fumigatus* isolates (systematic public health surveillance for *A. fumigatus* has not been conducted in the United States). In 2016, CDC received the first TR_34_/L98H isolate through this passive monitoring system, and an additional four have been identified to date among approximately 2,300 total isolates received (*6*). Together, these five and the two previously reported isolates (*5*) represent the first seven TR_34_/L98H isolates identified in the United States (Table). This report provides epidemiologic and clinical descriptions of the patients associated with these *A. fumigatus* triazole-resistant isolates.

**TABLE:** Characteristics of seven patients from whom TR_34_/L98H triazole-resistant *Aspergillus fumigatus* was isolated — California, Pennsylvania, and Virginia, 2010–2017

State of origin	Collection year	Source	Cyp51 genotype	Age range (yrs)	Sex	Underlying disease	Known previous triazole exposure?	Previous triazole exposure description	Colonization versus infection (suspected)*	Antifungal treatment	Outcome
Pennsylvania^†^	2010	Sputum	TR_34_/L98H	20–29	F	Respiratory failure following stem cell transplant	Yes	VRC; dose and duration unknown	Infection	VRC and CAS; L-AmB and CAS	Died
Pennsylvania^†^	2014	BAL	TR_34_/L98H	40–49	M	*A. fumigatus* colonization following lung transplant that progressed to multifactorial pneumonia and clinical IPA	Yes	VRC, ITC; dose and duration unknown	Infection	ITC and CAS; POS and CAS; L-AmB and CAS	Died
Pennsylvania	2016	Sputum	TR_34_/L98H	60–69	F	Chronic IPA, sarcoidosis	Yes	VRC 200 mg/day; duration unknown	Infection	VRC; CAS	Alive at discharge
Pennsylvania	2017	BAL	TR_34_/L98H	80–89	F	Hydropneumothorax with history of COPD and pulmonary fibrosis	No	Inpatient hospitalization, primary care, pulmonologist and pharmacy records indicate no record of triazole or other antifungal prescriptions	Colonization	None	Died
Virginia (nonresident)	2016	Sputum	TR_34_/L98H	70–79	M	Acute bronchitis and lung nodules; no history of immunocompromise	No	No triazole history available or suspected before hospitalization in Virginia; patient resides in Guatemala	Colonization	None	Alive at discharge
Virginia	2016	Sputum	TR_34_/L98H	20–29	F	Cystic fibrosis	No	None reported in 6 months preceding isolate collection	Colonization	None	Alive at discharge
California	2017	Sputum	TR_34_/L98H	80–89	F	COPD, chronic heart failure, and chronic kidney disease	No	No triazole history available or suspected before hospitalization	Colonization	None	Alive at discharge

**Abbreviations:** BAL = bronchoalveolar lavage; CAS = caspofungin; COPD = chronic obstructive pulmonary disease; F = female; IPA = invasive pulmonary aspergillosis; ITC = itraconazole; L-AmB = liposomal amphotericin B; M = male; POS = posaconazole; VRC = voriconazole.

* Colonization versus infection indicated based on explicit description in patient medical record or by treating physician, or, if not explicitly stated, suspicion based on public health review of record.

^†^ Wiederhold NP, Gil VG, Gutierrez F, et al. First detection of TR_34_ L98H and TR_46_ Y121F T289A Cyp51 mutations in *Aspergillus fumigatus* isolates in the United States. J Clin Microbiol 2016;54:168–71.

## Clinical Summaries

**Pennsylvania, 2010.** Following stem cell transplantation for sickle cell anemia, a woman developed graft-versus-host disease and respiratory failure. Resistant *A. fumigatus* was isolated from sputum. Despite therapy with voriconazole and caspofungin, her respiratory status worsened, and therapy was switched to amphotericin B and caspofungin. She deteriorated further and died of multisystem organ failure 6 months after isolate collection.

**Pennsylvania, 2014.** A man with *A. fumigatus* colonization following lung transplantation initially was treated with long-term voriconazole followed by itraconazole. He was hospitalized with bacterial and viral pneumonia, developed clinical invasive pulmonary aspergillosis, and was treated with itraconazole and caspofungin, followed by posaconazole and caspofungin, then inhaled amphotericin B. Resistant *A. fumigatus* was isolated from a bronchoalveolar lavage. With worsening clinical status and persistently positive *A. fumigatus* cultures, therapy was switched to liposomal amphotericin B and caspofungin; however, bronchoscopy indicated ongoing fungal infection. He died from multisystem organ failure approximately 2 months after isolate collection.

**Pennsylvania, 2016.** A woman with sarcoidosis and invasive pulmonary aspergillosis was treated with low-dose voriconazole because of vision-associated side effects at higher doses. Respiratory symptoms had worsened at the time of sputum collection, and when the resistant *A. fumigatus* isolate was identified, therapy was changed to caspofungin for 12 months. Following therapy, the patient was clinically stable with no radiographic evidence of progression to chronic cavitary pulmonary aspergillosis or aspergilloma.

**Pennsylvania, 2017.** A resistant *A. fumigatus* isolate was collected by bronchoalveolar lavage from a woman with chronic obstructive pulmonary disease, interstitial pulmonary fibrosis, and hypersensitivity pneumonitis, while she was hospitalized for hydropneumothorax and bacterial pneumonia secondary to trauma; no antifungal treatment was given. The patient died of complications of her hydropneumothorax thought to be unrelated to *A. fumigatus*.

**Virginia, 2016, case 1.** A man who visited Virginia from Guatemala was hospitalized for acute bronchitis 3 weeks after his arrival. Resistant *A. fumigatus* was isolated from sputum during this hospitalization. No antifungals were administered, and the patient was discharged to primary care.

**Virginia, 2016, case 2.** A woman with cystic fibrosis had resistant *A. fumigatus* isolated from sputum at an outpatient visit 2 days before hospital admission for a cystic fibrosis exacerbation. While hospitalized, she received steroids and antibiotics but not antifungals. She was later discharged with oral antibiotics.

**California 2017.** A woman with a history of chronic obstructive pulmonary disease requiring inhaled corticosteroids, chronic heart failure, and chronic kidney disease was evaluated as an outpatient for a productive cough. Sputum cultures grew *A. fumigatus*, and IgG antibody to *A. fumigatus* was twice the normal value. She was not started on antibiotics or antifungals.

## Discussion

*A. fumigatus* strains with mutations conferring resistance to mold-active triazole agents have been found in clinical and environmental specimens in the United States. In total, 10 U.S. clinical isolates with these genotypes (seven TR_34_/L98H and three TR_46_/Y121F/T289A) have been reported (*5*,*10*). Together, these reports likely underrepresent the number of U.S. isolates because aspergillosis and *A. fumigatus* colonization are not reportable in any state and few laboratories perform susceptibility testing for *Aspergillus* species. Four of the seven patients with TR_34_/L98H were not treated with antifungal therapy following culture; these four isolates, all from sputum or bronchoalveolar lavage, likely reflected *A. fumigatus* colonization rather than infection. However, the presence of highly resistant *A. fumigatus* strains in patient isolates suggests that U.S. clinicians need to be aware of the risk for triazole-resistant aspergillosis. Notably, four patients had no known exposure to antifungal medications before culture of the resistant isolate, supporting possible environmentally acquired resistance.

The five isolates identified at CDC during 2016–2017 were collected from patients who did not share health care facilities, procedures, or county of residence, arguing against shared health care acquisition. Given that *A. fumigatus* can undergo selection for antifungal resistance during triazole fungicide exposure in the environment, and spores of resistant strains might be transmitted through the air and inhaled, further exploration of triazole fungicide use and presence of triazole-resistant *A. fumigatus* in these areas is warranted.

The findings in this report are subject to at least two limitations. First, among the seven *A. fumigatus* isolates with the TR_34_/L98H mutations identified in the United States to date, four were collected in Pennsylvania, two in Virginia, and one in California. These three states contributed only 28% of all CDC *A. fumigatus* isolates collected during 2015–2017, raising the possibility of geographic localization. Second, because isolates were collected through passive monitoring and not systematic surveillance, caution must be exercised when interpreting these findings.

With environmentally derived TR_34_/L98H triazole-resistant *A. fumigatus* detected in the United States, systematic surveillance, detailed geographic data, and data on triazole fungicide use could be important for assessing the scope of the problem and trends in resistance. Exploration of risk factors for patient acquisition might provide opportunities to prevent exposure and mitigate risk for invasive infection in susceptible populations. Clinicians and microbiologists need to be aware of the possibility of triazole-resistant *A. fumigatus* infections, even in triazole-naïve patients. Expanded capacity to test for antifungal susceptibility in *A. fumigatus* could help inform clinical and public health decisions.

**Summary**

**What is already known about this topic?**

The environmental mold *Aspergillus fumigatus* is the primary cause of invasive aspergillosis. In patients with high-risk conditions, mortality exceeds 50%.* A. fumigatus* isolates resistant to medical triazoles have recently been identified in the United States in clinical and environmental specimens. The resistance marker TR_34_/L98H causes resistance to all triazoles and is associated with agricultural and environmental fungicide use.

**What is added by this report?**

Seven U.S. clinical TR_34_/L98H *A. fumigatus* isolates were identified during 2010–2017 from three states; four were collected from patients with no known previous triazole exposure.

**What are the implications for public health practice?**

U.S. clinicians and public health personnel should be aware that infections with triazole-resistant *A. fumigatus* can occur in patients not previously exposed to these medications.
